# Supplementary material for: Longitudinal genomic surveillance of Plasmodium falciparum malaria parasites reveals complex genomic architecture of emerging artemisinin resistance
Source: Genome Biol. 2017 Apr 28;18:78. doi: 10.1186/s13059-017-1204-4 (PMC5410087; doi:10.1186/s13059-017-1204-4)

Figure S1 – **Distribution of clearance rate half-life of samples exhibiting wildtype and mutant *kelch13*.** Box boundaries represent the first and third quartiles and the length of whiskers corresponds to 1.5 times the interquartile range.

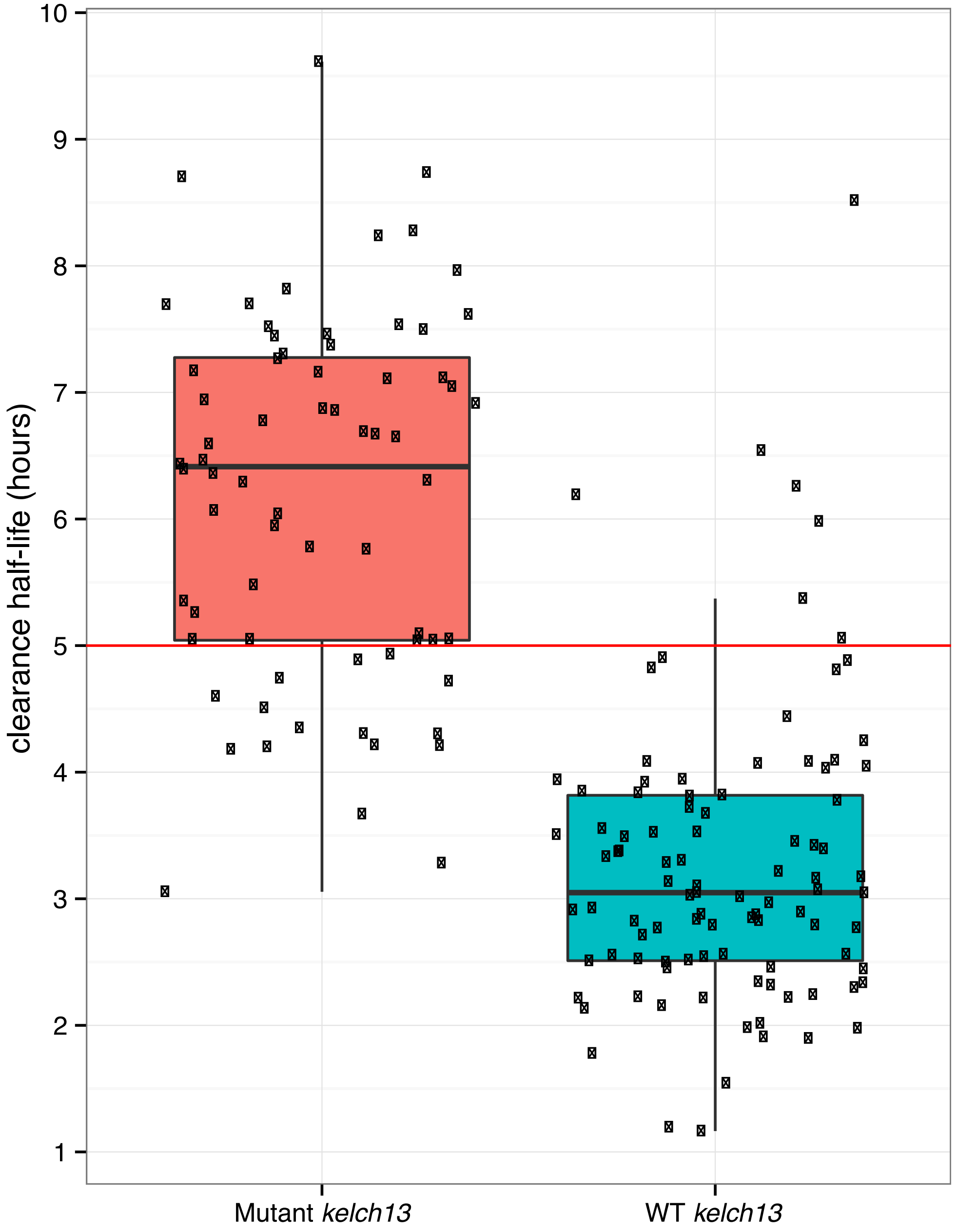

Figure S2 – **Non-reference allele frequency (NRAF) for *kelch13* polymorphisms.** (A) Colored vertical bars represent the NRAF of each *kelch13* mutation detected in our dataset and listed on panel B. Diagonal lines connect the boundaries of the bars representing the same mutation across the three sampling intervals. (B) List of the NRAF of each *kelch13* mutation and the NRAF of "mutant *kelch13*" (sum of the NRAF of all *kelch13* mutations) observed during the four sampling intervals.

A

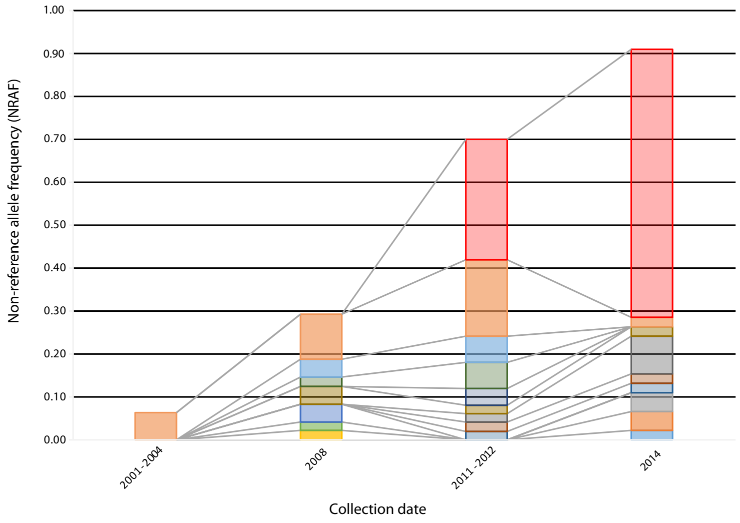

B

Non-reference allele frequency (NRAF)

	2001 - 2004	2008	2011 - 2012	2014
C580Y	0.00	0.00	0.28	0.62
E252Q	0.06	0.10	0.18	0.02
A675V	0.00	0.04	0.06	0.00
G538V	0.00	0.02	0.06	0.00
N537I	0.00	0.00	0.04	0.00
P441L	0.00	0.04	0.02	0.02
N458Y	0.00	0.00	0.02	0.09
F446I	0.00	0.00	0.02	0.02
R561H	0.00	0.00	0.02	0.02
P574L	0.00	0.04	0.00	0.00
M476I	0.00	0.02	0.00	0.00
S485N	0.00	0.02	0.00	0.00
P553L	0.00	0.00	0.00	0.04
G533A	0.00	0.00	0.00	0.04
P667T	0.00	0.00	0.00	0.02

Figure S3 – **Location of *kelch13* and *kelch10* mutations and clearance half-life of samples harboring those mutations**. (A) Location of SNPs on *kelch13* gene (horizontal axis) and clearance half-life of each sample harboring those mutations (vertical axis). (B) Location of InterPro domains identified on *kelch13* and *kelch10* amino acid sequence. Blue projections indicate similar regions according to BLAST. The position of P623T mutation on *kelch10* is indicated by a black diamond. (C) Results of BLAST similarity search between *kelch13* (query) and *kelch10* (subject) amino acid sequence. (D) Box plots showing the distribution of clearance half-life of samples harboring wild-type (WT) and mutant *kelch13* (affected amino-acid listed on the bottom) and either with (green) or without (white) P623T *kelch10* mutation. Box boundaries represent the first and third quartiles and the length of whiskers corresponds to 1.5 times the interquartile range.

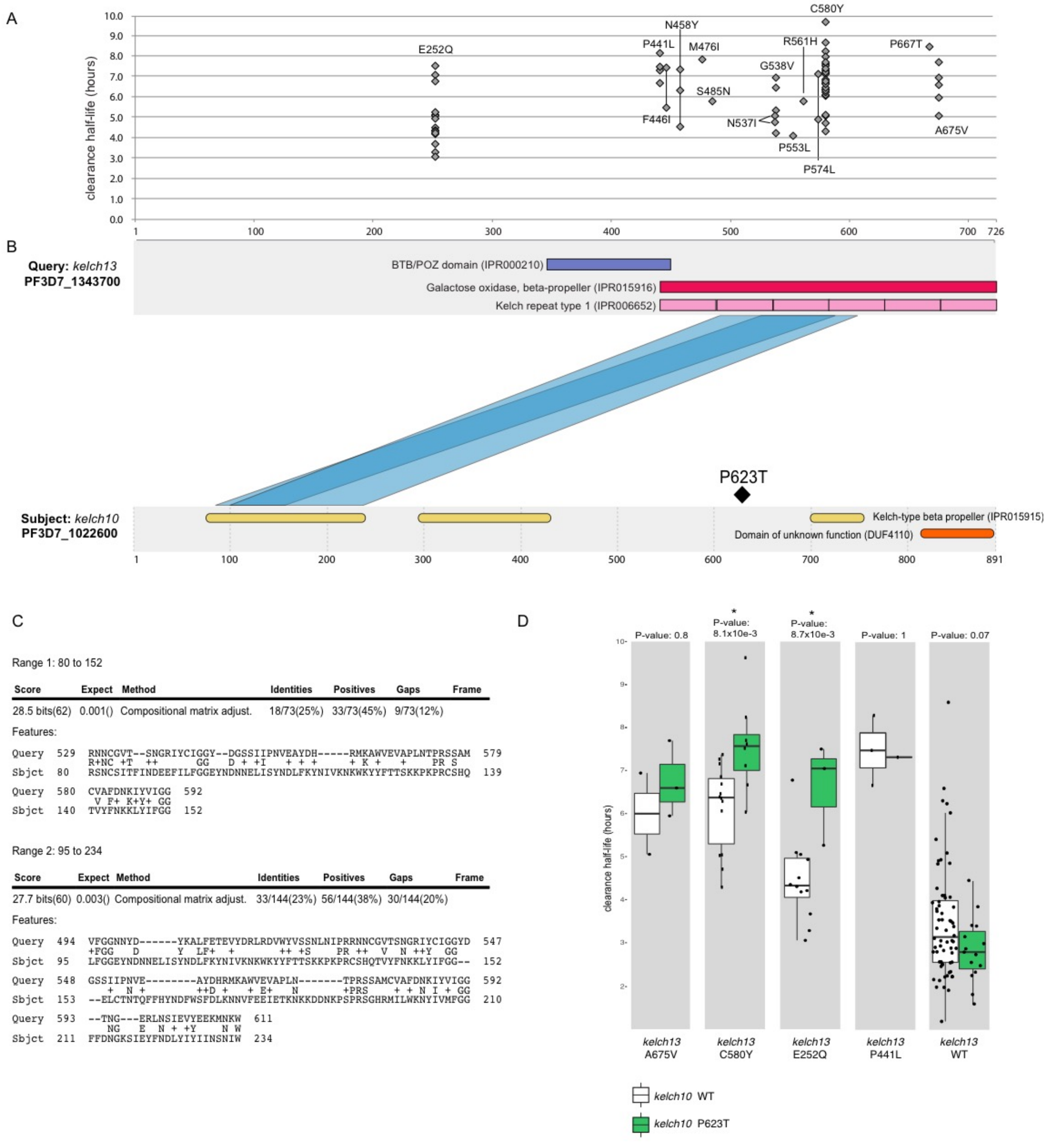

Figure S4 – **Evidence of two independent origins of the C580Y allele.** Rows represent a subset of the samples in our dataset harboring a C580Y mutation. Columns represent all polymorphic sites in the vicinity of C580Y allele. Colored circles indicate the genotype according to the legend on the left. Plus signs indicate positions with distinct genotypes between the two haplotypes blocks.

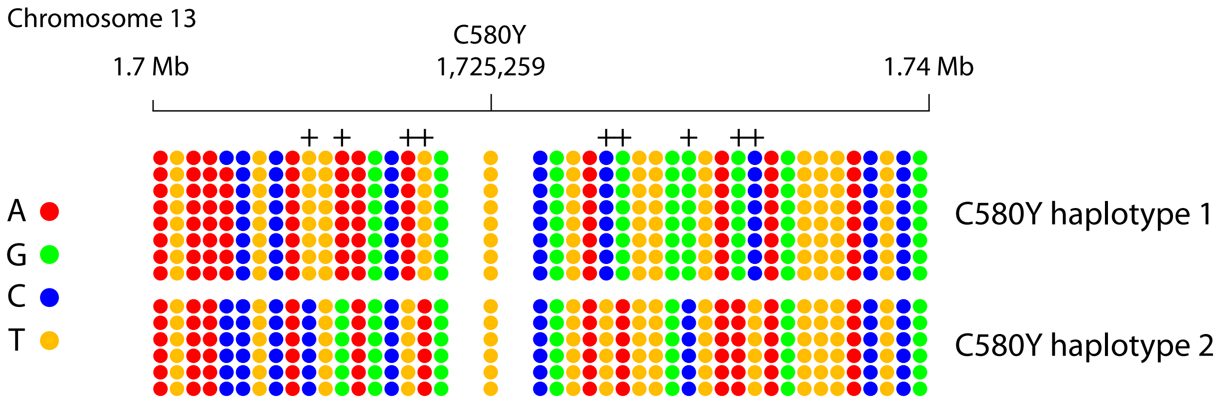

Figure S5 – **Results of the GWAS for clearance rate.** A) Q-Q plot comparing the expected distribution of p-values and the observed distribution based on the Wald test implemented by the GEMMA package [65]. C580Y has the lowest p-value among all SNPs evaluated. B) Manhattan plot indicating the location of SNPs along the 14 nuclear chromosomes of *P. falciparum*.

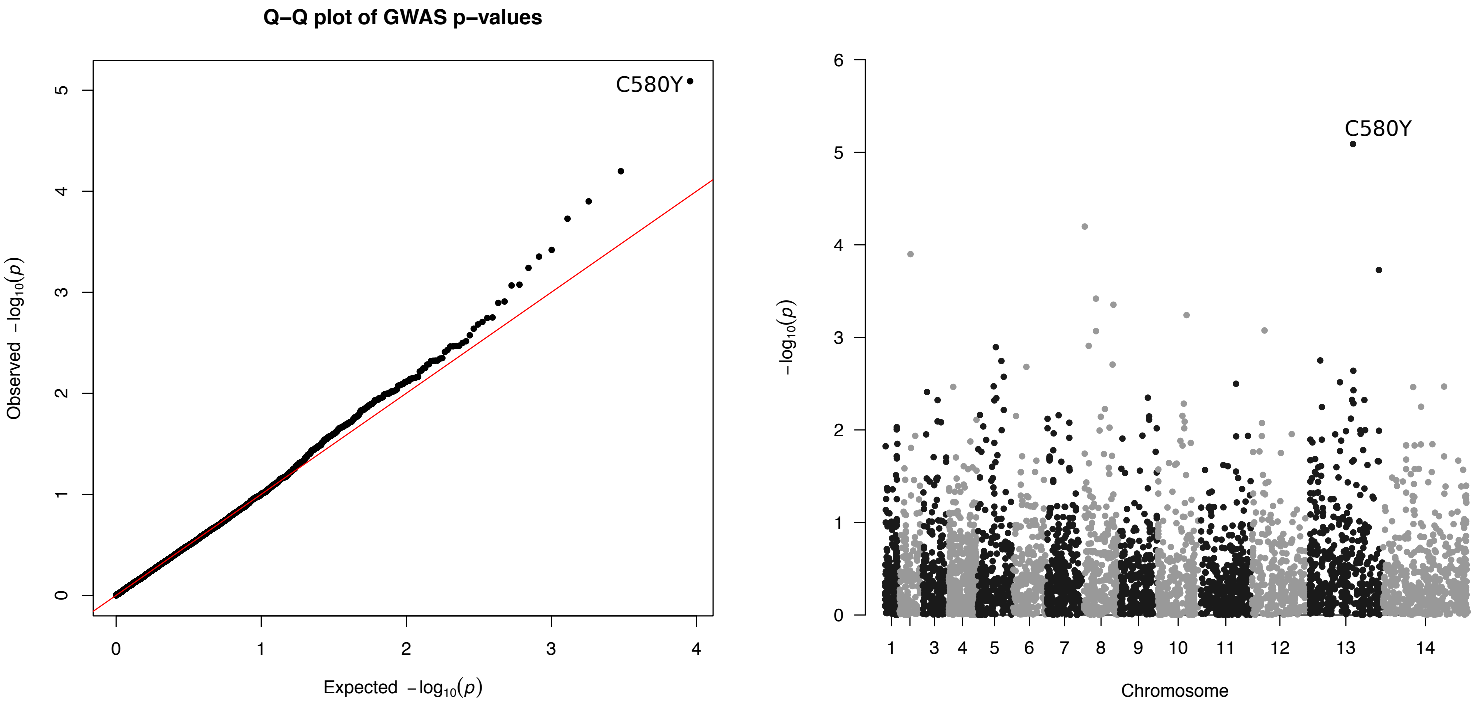

Figure S6 – **Selection on C580Y-vector-like candidate SNPs.** (A) Graph indicating the slope of the ranked distance between C580Y vector and others SNPs in the dataset (see Materials and Methods for details). Only the 500 closest SNP-vectors to C580Y-vector are shown. Rectangle in red indicates a contiguous stretch of 30 data points with slope equal to 0. (B) Trajectory of the allele frequency of C580Y-vector-like SNPs across the first three time intervals. (C) Distribution of linkage disequilibrium (*r*) between mutant *kelch13* and C580Y-vector-like (black) and control SNPs (blue), binned according to the 2011-2012 NRAF (interval left-closed, right-open). Box boundaries represent the first and third quartiles and the length of whiskers corresponds to 1.5 times the interquartile range. Controls SNPs have comparable NRAF in 2011-2012, but exhibit non-zero NRAF in the earlier collection phases. P-values indicate significantly different distributions between C580Y-like and control SNPs (Wilcoxon test). (D) Comparison of the distribution of non-normalized iHS values for C580Y-vector-like SNPs and control SNPs. Box boundaries represent the first and third quartiles and the length of whiskers corresponds to 1.5 times the interquartile range. Lower iHS values indicate stronger signature of selection. P-values on the top of the graph indicate significantly different distributions of C580Y-like and control SNPs (Wilcoxon rank sum test).

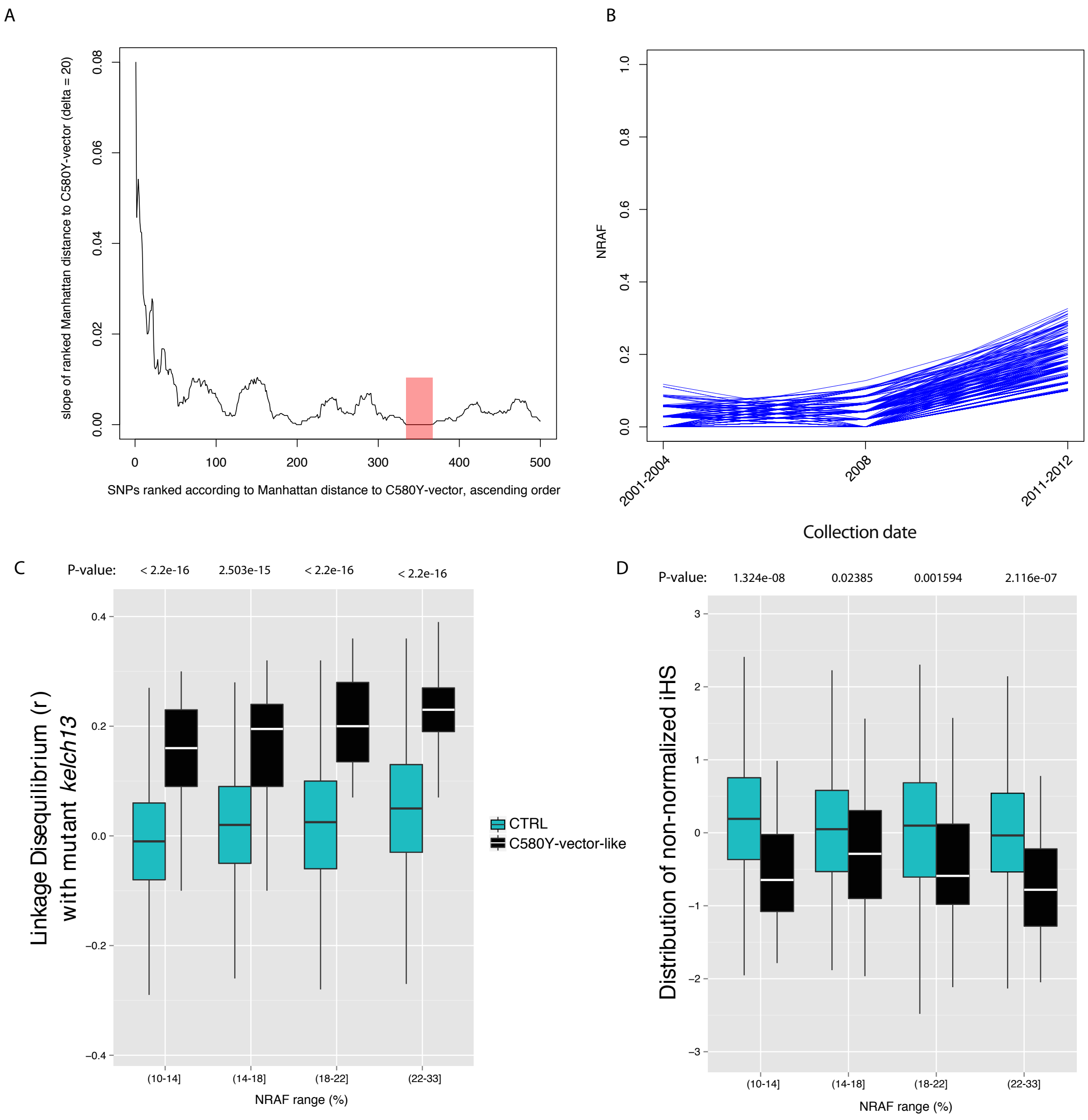

Figure S7 – **Pairwise identity by descent along the genome.** (A) Distribution of candidate SNPs along the *P. falciparum* genome. Black and red dots denote mutations identified as part of the C580Y-like and/or C580Y-vector-like sets and the P623T *kelch10* mutation identified by GWAS (see Results section). Horizontal axis indicates the position of SNPs along each chromosome, labeled on the bottom of the panel. Vertical axis indicates the value of Shannon entropy index of the association with distinct *kelch13* mutations (see Methods section). (B) Incidence of pairwise isolate comparisons exhibiting identity by descent (IBD) along the genome Isolates from all four time points were included in this analysis. The locations of the *kelch13* C580Y mutation, *kelch10* P623T mutation, and PI4K mutation are indicated by red dots.

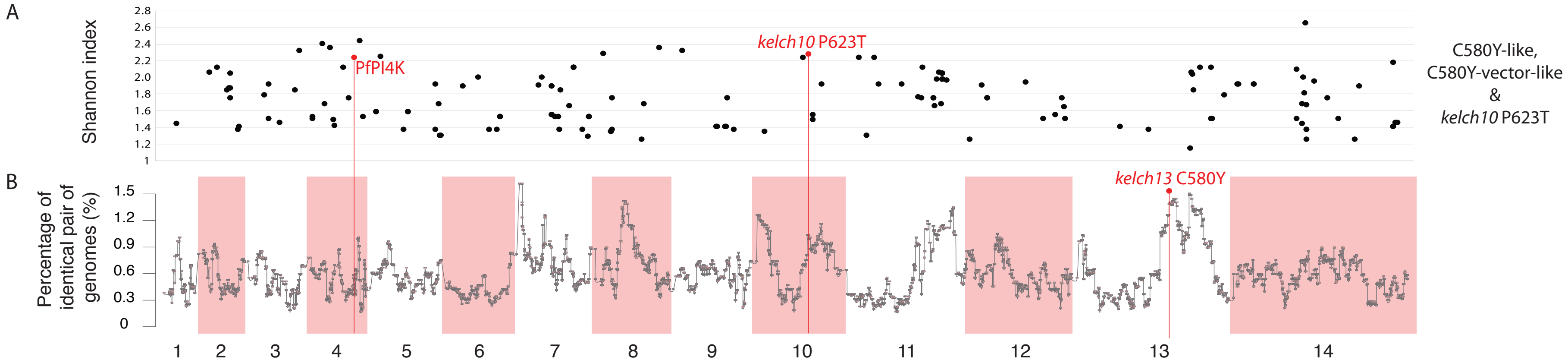

Supplement: Supplementary file 1 — (PDF 3253 kb) [file 13059_2017_1204_MOESM1_ESM.pdf]
